# Radiotherapy in Uveal Melanoma: A Review of Ocular Complications

**DOI:** 10.3390/curroncol30070470

**Published:** 2023-07-03

**Authors:** Lamprini Banou, Zoi Tsani, Konstantinos Arvanitogiannis, Maria Pavlaki, Anna Dastiridou, Sofia Androudi

**Affiliations:** Department of Ophthalmology, University of Thessaly, 41110 Larissa, Greece; labrinibanou@gmail.com (L.B.); zoi.tsani@outlook.com (Z.T.); arvanitoyiannis@gmail.com (K.A.); pavlaki.em@gmail.com (M.P.); adastirid@uth.gr (A.D.)

**Keywords:** uveal melanoma, radiotherapy, ocular complications

## Abstract

Uveal melanoma represents the most prevalent form of primary malignant intraocular tumor in adults. Historically, enucleation was considered the gold-standard approach in the treatment of uveal melanoma. Currently, radiotherapy is the most commonly used therapy, aiming at a better quality of life. However, radiotherapy can result in several ocular complications, some of which may be vision-threatening. Radiation-induced dry eye, scleral necrosis, cataract, rubeosis iridis, neovascular glaucoma, radiation retinopathy, maculopathy, and optic neuropathy are the most common complications. This article aims to summarize the current literature regarding the ocular complications after radiotherapy, as well as their clinical features, risk factors, and management strategies. A thorough understanding of these issues is crucial for ophthalmologists and oncologists to provide optimal patient care, improve visual outcomes, and minimize long-term complications.

## 1. Introduction

Uveal melanoma (UM) represents the most common malignant intraocular tumor, with almost 90% being located in the choroid. Historically, enucleation was considered the gold-standard approach in the treatment of uveal melanoma. However, after the Collaborative Ocular Melanoma Study (COMS) demonstrated that there was no survival advantage between the enucleation and radiotherapy group, the focus was shifted, whenever feasible, towards globe-preserving therapies, aiming at a better quality of life [1,2,3]. Proton beam radiotherapy (PBT), plaque brachytherapy, and stereotactic radiotherapy and radiosurgery are currently used successfully. Radiotherapy is a valuable eye- and vision-preserving tool; however, many patients may suffer complications, some of which can be sight-threatening. Various complications may occur, including radiation-induced dry eye, scleral necrosis, cataract, rubeosis iridis, neovascular glaucoma, retinopathy, and optic neuropathy [4]. The complications may be tumor-related or radiation-induced. The goal is to improve understanding and awareness of these complications, highlighting the importance of early diagnosis, the establishment of a treatment plan, and expert medical care, when necessary.

## 2. Materials and Methods

Using PubMed, a literature search was conducted with the terms: “uveal melanoma”, “radiotherapy”, “plaque brachytherapy”, “proton beam radiotherapy”, “stereotactic radiotherapy”, “ocular complications”, and “management”. Articles published up until March 2023 were reviewed. The retrieved articles were assessed for eligibility and filtered manually to exclude duplicates, while articles cited in the reference lists were reviewed to ensure that no relevant studies were overlooked. Articles in the English language were included.

## 3. Results

### 3.1. Uveal Melanoma

Uveal melanoma (UM) is the most common primary intraocular malignancy in adults, accounting for 5% of all melanomas. UMs arise from melanocytes in the pigmented uveal tissues of the eye, which consists of the iris, ciliary body, and choroid [2,5,6]. Nearly 90% of UMs involve the choroid, with only 6% being located at the ciliary body and 4% at the iris. Despite treatment of the primary tumor, studies have found that approximately 50% of patients will develop metastasis, often through hematogenous spread, with the liver being the first site of metastasis in 90% of the cases [2,5,6]. The incidence of uveal melanoma in the United States is 5 per million people, while in Europe it ranges from 2 to 8 per million people, following a north-to-south decreasing gradient [7,8,9]. The disease primarily affects individuals between 50 and 70 years old, usually unilaterally [2].

Risk factors for the development of uveal melanoma include fair skin, light eye color (green or blue), welding, congenital ocular melanocytosis, dysplastic nevi, and *BAP1*- tumor predisposition syndrome [2,10]. UM is characterized by a very low mutation burden [5,11]. Although there are numerous factors linked to prognosis, tumor size remains the most critical clinical factor affecting prognosis, with larger and thicker tumors being linked to a worse outcome. Older age at presentation of the disease, male gender, the association with ciliary body involvement, extrascleral extension, certain histopathologic and cytogenetic features, and an advanced staging according to the American Joint Committee on Cancer (AJCC) can also contribute to a poor prognosis [6,7,12]. AJCC staging is based on the tumor size, the extent of spread to the regional lymph nodes, and the presence of distant metastasis (Tumor-Node-Metastasis-TNM staging system) [6,13]. AJCC also includes the genomic profile of the tumor in their prognostic factors. Chromosomal alterations, such as chromosome 3 status (loss or no loss, complete or partial) and chromosome 6p and 8q status (gain or no gain), may influence the prognosis. Moreover, the gene expression profile (class 1 or class 2) may play an important role in the prognosis [13].

### 3.2. Clinical Presentation—Diagnosis

Most patients with uveal melanoma present with blurred or distorted vision, visual field loss, or photopsia, but about 30% remain asymptomatic and are diagnosed during a routine examination [2,14,15]. The diagnosis of choroidal melanoma is based primarily on a detailed fundus evaluation with slit-lamp biomicroscopy and indirect ophthalmoscopy [9,14], but several other techniques are available to improve the diagnosis of UM, including imaging tools such as optical coherence tomography, ultrasound microscopy, and UBM [15]. Magnetic resonance imaging (MRI) is also a valuable tool used for the detection, characterization, and evaluation of the local extent of the disease, as well as for monitoring treatment-related complications and follow-up [15]. It is particularly useful in patients with opaque media [15]. The accuracy of clinical diagnosis is high when conducted in reference centers [15], and the Collaborative Ocular Melanoma Study (COMS) reported a greater than 99% diagnostic accuracy for the enucleation eyes with typical features [16]. Biopsy is rarely necessary and is used when clinical examination and imaging are inconclusive [2,9,14]. Patients with suspicious pigmented lesions should be assessed by an ophthalmologist with clinical expertise in ocular tumors [14]. Differential diagnosis is also important, as a variety of lesions can simulate posterior uveal melanoma [9].

### 3.3. Treatment

Historically, enucleation was considered the gold-standard for the treatment of uveal melanoma. However, after the Collaborative Ocular Melanoma Study demonstrated that radiotherapy was equally effective, the focus was shifted, whenever feasible, towards globe-preserving therapies [1,2,3]. Currently, the most widely used treatment method is radiation therapy.

Radiation therapy is designed to damage the DNA of the cancer cells in order to prevent them from dividing and continuing to grow [17,18,19]. However, radiation can also damage the DNA of normal cells, which can lead to side effects. The goal of radiation therapy is to carefully target the cancer cells while minimizing damage to nearby healthy cells. This is achieved by carefully planning the radiation treatment, using sophisticated imaging techniques to precisely target the tumor, and using special equipment to deliver the radiation from different angles. By using these techniques, radiation oncologists are able to maximize the dose of radiation to the cancer cells while minimizing the dose to healthy cells, which can help reduce side effects and improve treatment outcomes [17,18]. proton beam radiotherapy, plaque brachytherapy (Iodine-125, Ruthenium-106, Palladium-103, Cesium-131), and stereotactic radiotherapy and radiosurgery (Gamma Knife, CyberKnife, LINAC) are currently used successfully.

Treatment plans should always be customized, taking into account various factors, such as the lesion’s location, its size, the condition of the unaffected eye, as well as the physical and psychological condition, needs, and preferences of the patient [1,19].

### 3.4. Ocular Complications after Radiation Therapy

Radiotherapy can affect almost every structure of the eye, resulting in more or less severe side effects that require management. The ocular complications following radiotherapy are analyzed according to the structure affected. Moreover, the therapeutic approach to each complication is summarized in Table 1. In Table 2, we refer to the current clinical trials in the prevention or treatment of ocular complications after radiotherapy.

#### 3.4.1. Ocular Surface and Ocular Adnexa Complications

**Figure 1:**
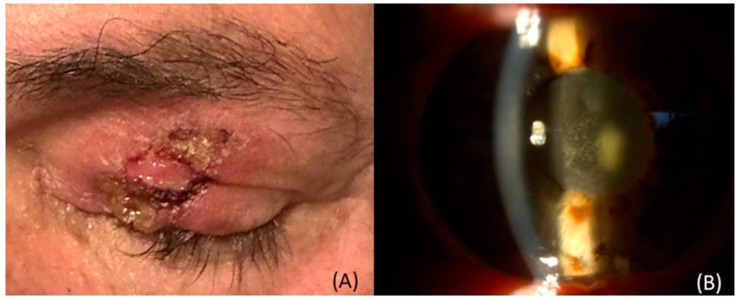
(**A**) Severe dermatitis post-radiation. (**B**) Cataract formation after radiotherapy.

Possible adverse effects on the surface of the eye can range from minor issues, like transient dryness or inflammation, to more severe conditions, such as corneal disintegration and piercing, also comprising skin blemishes and discoloration, loss of eyelashes, with associated scarring, and abnormal tissue change in the conjunctiva and edges of the eyelids (Figure 1) [70]. This could lead to inflammation and scarring of the tear ducts and canals, resulting in continual excessive tear production. Meanwhile, radiation of the tear gland causing its subsequent shrinkage could lead to a type of dry eye syndrome known as keratoconjunctivitis sicca, particularly in tumors located near the temples [70].

The adverse outcomes of proton beam therapy (PBT) are chiefly determined by the tumor’s magnitude and position, with diminished corneal sensitivity, potentially escalating to full numbness, which often serves as an early sign of radiation-induced corneal damage [21]. A decrease in the corneal nerve supply disrupts both defensive reflexes and essential cell regulators, causing painless ulcers on the central or edge of the cornea due to a lack of limbal stem cells, a condition that occurs in approximately one-third of patients receiving radiation treatment for the front part of the eye [71]. Proton beam radiation (PBT) can also influence the upper eyelid and the tear drainage system, potentially leading to conditions like inflammation of the tear duct or blockage of the tear duct opening [51,72].

Compared to charged particle radiotherapy, brachytherapy results in fewer complications in the front part of the eye [73]. Ruthenium plaques rarely result in complications in the anterior segment; however, conditions like conjunctival separation and scleral decay have been observed, especially when the conjunctival closure in the area of muscle detachment is inadequate [74]. Up until now, no significant ocular surface damage has been reported following treatment with the Cyber-Knife system. However, the available literature on this technique remains quite limited [75,76,77].

#### 3.4.2. Sclera

Scleral complications after radiotherapy are uncommon because of the nature of this avascular, hypocellular tissue, which is remarkably radioresistant [22,27]. Scleral necrosis, corneoscleral necrosis, and scleritis are the most addressed post-radiation complications of the sclera. Jones and Reese were the first to report scleral necrosis after gamma or beta radiation, followed by reports of scleral necrosis after 60Co (2–9%), 192Ir (1%), 106Ru (0–9%), 198Au (12%), 125I (0–11%), and proton beam radiotherapy (1%) for uveal melanoma [22,23,24,25,26,27,28,78,79]. The comparative incidence of scleral necrosis with each radionuclide is not clearly understood. According to Kaliki et al., 73 patients out of 5057 (1%) treated with plaque therapy (125I, 60Co, 192Ir, or 106RU) for ocular melanoma presented scleral necrosis as a post-radiation complication in a mean follow-up period of 79 months [22]. In addition, Radin et al. reported 23 patients with scleral necrosis after plaque radiotherapy with 60Co or I125 (1.5% of treated patients) or proton beam radiotherapy (0.45% of treated patients) [23]. In 2002, Shields et al. reported scleral necrosis in 7% at 5 years and in 9% at 10 years post-treatment in 354 consecutive patients treated with plaque radiotherapy (iodine 125) for large posterior uveal melanomas (≥8 mm thickness). On the other hand, the proportion of scleral necrosis was even higher in 136 patients with ciliary body melanoma after receiving 125I plaque treatment, reaching 11% in a median follow-up of 70 months [24]. Chaudry et al. reported 15 patients (7.4%) with symptomatic corneoscleral necrosis after therapy with Au-198 radioactive plaque, while corneoscleral necrosis has also been reported after beta irradiation following pterygium excision [28].

The mean time of onset of scleral necrosis after radiation therapy ranges from 27 to 70.4 months, although there are a few case reports of early conjunctival and scleral necrosis in the postoperative period, when the mean scleral necrosis diameter at its onset is 4 to 4.4 mm [22,23,26]. Scleral necrosis is characterized by an almost total absence of any symptoms, unless it involves the cornea, and it is occasionally accompanied by overlying pigmentation deposits, rendering its recognition, its differentiation from relapse of previously treated uveal melanoma with extrascleral extension, and its management difficult [22,23,27,28]. Distinguishing scleral thinning from recurrence relies on key indicators such as the utilization of ultrasound biomicroscopy or anterior segment optical coherence tomography to detect thinning, the presence of blue discoloration in the Tenon’s capsule, increased transillumination, and a progressive decrease in intraocular melanoma thickness over time [22]. Detecting posterior necrosis may be challenging during routine funduscopic examinations, requiring a high level of suspicion based on other clinical manifestations such as extreme hypotony and unexplained vision loss to confirm the diagnosis [27].

The primary causes of scleral necrosis involve various mechanisms, including the direct damaging effect of radiation on the sclera, the indirect consequence of the disinsertion of the extraocular ocular muscles leading to local ischemia, inflammation associated with tumor necrosis, regression of tumors with concealed invasion into the sclera, or an underlying systemic autoimmune phenomenon that may not be readily evident [23]. Predictive factors of evident scleral necrosis in the clinical setting include the anterior location of the tumor margin, more specifically, the ciliary body and pars plana to ora serrata, the size of the tumor ≥6 mm, high radiation dose (≥400 Gy) to the outer sclera, and higher intraocular pressure (>15 mmHg) [22,23,24,25,26]. As mentioned previously, the posterior location of scleral necrosis after radiation therapy may be underdiagnosed, as it cannot be easily located by external examination and ultrasonography, affording the anterior location of the tumor with greater rates of this complication [27]. Radin et al. correlated scleral necrosis with a higher incidence of cataract, retinopathy, and maculopathy, as a result of higher radiation doses due to larger tumors [23].

Scleral necrosis is not typically an eye-threatening complication, unless perforation occurs, which reportedly happens in 4–8.5% of patients [22,23]. Treatment of scleral necrosis includes observation the majority of patients, as in nearly half of them, it will remain stable [22]. Depending on the severity of scleral and corneoscleral necrosis, there are various management options (Table 1). These include artificial lubrication with tears, gels, or ointments; prednisolone acetate 1% for treatment of early onset scleral necrosis; tissue glue; conjunctival/flap graft; amniotic membrane transplantation; scleral patch graft; dermal patch graft; vital Tenon’s fascia transposition; hyperbaric oxygen therapy; and enucleation [22,23,24,25,26,27,28].

#### 3.4.3. Lens and Cataract

The lens is the most radiation-sensitive tissue in the human body, and ionizing radiation in doses ≥ 10 Gy can result in the denaturation of heat-sensitive enzymes, distortion of the cellular DNA, and destruction of the pellucid lens cells through thermoelastic expansion [30,51,80]. Miguel et al. reported a 42% incidence of these complications in a 243 patient cohort over 20 years of monitoring at their center [39]. Posterior subcapsular cataracts are the most common type caused by radiation exposure, presenting as vacuoles and scattered granules, or in the case of larger tumors or higher radiation doses, as a mature white cataract [30,51,80]. The pace of cataract formation also varies greatly, as it depends on numerous factors, such tumor location, increased tumor height, older patient age, and the radiation dose received by the lens [51,81].

As the COMS trial demonstrated, the most prevalent side effect of plaque brachytherapy was radiation-induced cataract, which was the primary reason for diminished vision after treatment, (Figure 1), [1,82,83]. There was no discernible difference in post-treatment cataract rates among 125I, 103Pd, and 106Ru plaque types, but the size of the plaque, which is determined by tumor size, seems to affect the cataract formation rate [1,82,83]. At the 5-year follow-up, cumulative doses of <12 Gy, 12–15.9 Gy, 16–23.9 Gy, and >24 Gy were linked with 65%, 86%, 88%, and >92% cataract incidence, respectively, proving that a higher radiation dose correlated with an increased occurrence of lens opacity following the procedure [31,83]. Anterior and posterior tumors carried an 85% and 17% incidence of cataract, respectively, with the higher incidence rate being attributed to the lens’ anatomical proximity to the brachytherapy plaques in anteriorly-located tumors [31,83]. The rate of cataract development in PBT reportedly resembles that of radiation therapy [30,31,84,85]. Thariat et al. showed a dose-related cataract formation after PT and suggested that a lens-sparing approach may be feasible [86].

The primary goal of cataract surgery in uveal melanoma patients is to enable visualization of the fundus for funduscopic tumor control or posterior pole surgery (endoresection vitrectomy) and to a lesser extent, to improve visual acuity, which is often limited by radiation optic neuropathy and retinopathy [30,31,32]. Cataract surgery for these patients did not present a greater risk of complications, in comparison to cataracts not caused by radiation [30,82].

#### 3.4.4. Radiation Retinopathy

Radiation-induced retinopathy is a slowly progressive, delayed-onset disease of the retinal blood vessels, constituting the most common cause of permanent visual loss, especially when the macula is involved [27,37]. The disease occurs in about 25–28% of patients having been treated with PBT, while this percentage rise to 90% in a 5-year follow-up. The relative occurrence in patients treated with plaque radiotherapy is 4.6–66% within 20–31 months post-radiation, whereas it is around 17–35% after gamma knife radiosurgery [9,35,63,76,78,81,87,88,89,90,91,92,93,94,95,96,97,98]. According to Sagoo et al., approximately 66% of 650 eyes treated with plaque radiotherapy developed nonproliferative radiation retinopathy, while around 24% of eyes developed proliferative radiation retinopathy over 5 years of follow-up [99]. Histopathological findings typically include focal narrowing and the obliteration of capillaries, the disruption of pericytes and endothelial cells that form blood vessel walls, and the formation of microaneurysms [96]. These issues lead to a remodeling of blood flow, alternative channels with thickened and fenestrated walls, and irregular dilation of the adjacent microvasculature [27,37,96]. Eventually, capillary and pericyte loss occurs, followed by perivascular white sheathing. After a total occlusion, ghost vessels are seen, resulting in retinal ischemia and atrophy [37,96,98]. Patchy degeneration of the RPE in the form of loss of melanin, accumulation of lipofuscin, hyperplasia and beading, telangiectasia, microaneurysms, sclerosis, and closure of choroidal vessels have also been described [98].

Radiation retinopathy is a condition that manifests with retinal hemorrhages, microaneurysms, cottonwool spots, exudation, and microangiopathy within the retina [85,100]. This condition can also cause retinal ischemic changes, including capillary nonperfusion in the macula, infarcts in the nerve fiber layer, and neovascularization in the retina and optic nerve, as well as non-perfusion in the choriocapillaris and choroidal ischemia. [27,37,98]. In nearly all cases, the presence of microaneurysms is the first sign of radiation retinopathy that can be detected through ophthalmoscopy. [98]. The retinal hemorrhages are often absorbed, but can rarely progress to vitreous hemorrhage. Meanwhile, exudates may appear as soft white or cotton-wool spots at the early stages of treatment. These rapidly disappear, and hard exudates are more commonly seen. They may also be located in the macula in a star pattern, or appear to be similar to circinate retinopathy [37,96]. Ghost vessels can appear in the later stages of the disease. Radiation retinopathy progresses from non-proliferative to proliferative and can result in rapid deterioration of vision, with lower initial visual acuity and severe ischemic status being the major concerns [98].

Posterior location of the tumor, especially macular or peripapillary, high radiation rate (≥230 cGy/h), increased tumor thickness, and diabetes mellitus are the main risk factors for developing retinopathy, while older age and a mushroom configuration of the tumor seem to have a protective effect [22,24,37,85,98,99]. The presence of diabetes as a predictive factor suggests that eyes with preexisting vascular derangement are more susceptible to the development of radiation retinopathy [22].

In 2005, Finger and Kurli proposed a classification scheme for radiation retinopathy in order to characterize the prognosis for vision after radiotherapy: [37]

Stage 1: extramacular ischemic changes, good visual prognosis.

Stage 2: macular ischemic changes, moderate visual prognosis.

Stage 3: additional macular edema and extra-macular retinal neovascularization, low vision.

Stage 4: additional vitreous hemorrhage and at least five disc areas of retinal ischemia, low vision.

#### 3.4.5. Radiation Maculopathy

The macula is a radiosensitive tissue usually affected by radiotherapy. The incidence of radiation maculopathy after Ru-106 brachytherapy is 25.5–38% [81,91,101], after I125 plaque radiotherapy, it is 24.5% [92], after I125 plaque radiotherapy and adjunctive TTT laser, it is 38% [37], after palladium-103 plaque brachytherapy, it is 14–15% [71], after different types of plaque radiotherapy, it is 56–64% [87,99], after proton beam therapy, it is 23.8% [72], after gamma knife radiosurgery, it is 9–30% [93,94,97], and after fractionated stereotactic radiotherapy (fSRT), it is 23.8–81% [102,103,104]. Finger et al. [71] reported that among 400 patients treated with Palladium-103 plaque brachytherapy for uveal melanoma, approximately 14% developed radiation maculopathy, without optic neuropathy, while about 6% experienced both radiation maculopathy and optic neuropathy; there are no cases reported in which radiation-induced optic neuropathy occurred without maculopathy presenting simultaneously. On the other hand, Kim et al. noted that among 63 patients diagnosed with papillopathy, 73% of them were also diagnosed with radiation maculopathy after PBT [105].

**Figure 2:**
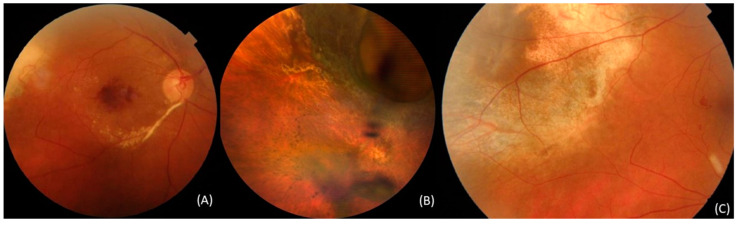
Fundus photos of patients after radiotherapy. (**A**) Radiation maculopathy; (**B**) post-radiation pigmentation and uveitis; (**C**) tumor-related lipid exudation (TRLE).

Radiation maculopathy is associated with the following: increased tumor size (thickness and greatest basal dimension), ≤2 mm distance between tumor margin and macula, tumor apex dose rate ≥80 cGy, the use of radioisotope Iridium-192 compared with Iodine-125, radiation dose to fovea >50 cGy, preexisting subretinal fluid, a dose delivered to more than 20% of the retina, diabetes mellitus, proximity of tumor to foveola, male gender, and younger age [34,35,36,37,81,87], (Figure 2).

Horgan et al. proposed a grading system using OCT for evaluating subretinal fluid.

Grade 1—extrafoveolar, non-cystoid edema;

Grade 2—extrafoveolar, cystoid edema;

Grade 3—foveolar, noncystoid edema;

Grade 4—mild to moderate foveolar cystoid edema;

Grade 5—severe foveolar cystoid edema.

They proposed that the final visual acuity of patients correlates with the grade of macular edema assessed by OCT at the time of onset and at the time of maximal macular edema, foveal thickness at onset of macular edema, and foveal thickness at the time of maximal macular edema [35].

McCannel et al. proposed another classification using fluorescein angiography [36].

Grade 0—no findings;

Grade 1—late foveal leakage;

Grade 2—late peripheral vascular and foveal leakage;

Grade 3—midphase nonperfusion (≥1 DA of lack of retinal vascular filling), late foveal and peripheral leakages;

Grade 4—retinal neovascularization, midphase nonperfusion, and late foveal and peripheral leakages.

Sub-tenon triamcinolone administration, intravitreal ranibizumab and bevacizumab injections every 2 months and 4 months, respectively, laser ablation of the ischemic peripheral retina, and sectoral peripheral laser treatment are proven to be effective prophylactic measures of radiation maculopathy [33,36,106,107,108]. The main adverse events of sub-tenon triamcinolone are ocular hypertension, cataract (30% to 45%), and prolapse of orbital fat [33,108]. Intravitreal anti-VEGF injection involves the risk of endophthalmitis and the theoretical risk of cardiovascular complications [87,108,109]. Radiation maculopathy is vastly suppressed by intravitreal anti-VEGF therapy, mainly through the use of bevacizumab, ranibizumab, and aflibercept [33,36,87,96,107,108]. Intravitreal delivery of corticosteroids, either triamcinolone acetonide or dexamethasone implant, is another potent treatment option [33,108]. The main adverse events include cataract formation and increased IOP. Singaravelu J et al. suggested intravitreal fluocinolone acetonide implant as an effective treatment, after observing the results from seven patients treated with CME radiation retinopathy [110].

#### 3.4.6. Retinal Detachment

**Figure 3:**
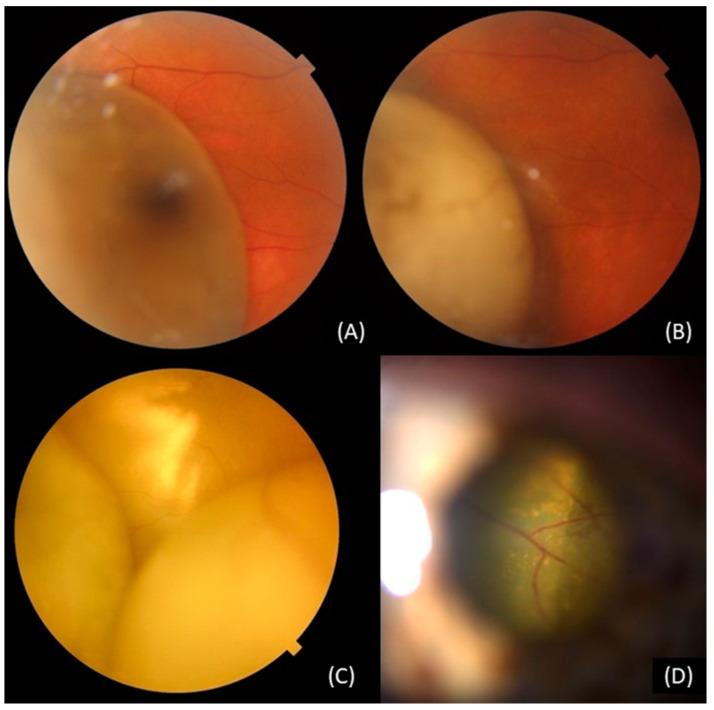
Fundus and slit-lamp photos of a patient after PBT: (**A**) choroidal melanoma at the time of the diagnosis; (**B**) at 7 months post-treatment; (**C**) exudative retinal detachment at 18 months post-treatment; (**D**) toxic tumor syndrome (RD, NVG, NVI).

Exudative retinal detachment (RD) is a very common occurrence in cases involving uveal melanoma, with the tumor base and apical height being closely related to the extent of the detachment, (Figure 3) [39]. Persistent exudation leads to three challenges in managing uveal melanoma, subsequently increasing the risk of local treatment failure. Firstly, it reduces the size of the vitreous cavity, restricting the amount of silicone oil that can be injected in the case of vitreoretinal surgery [111,112]. Silicone oil filling then hampers the examination using a B-scan. Secondly, it can lead to photoreceptor apoptosis and impaired vision, even if the macula is eventually reattached, due to persistent RD. Lastly, persistent exudative retinal detachment contributes to the development of proliferative vitreoretinopathy, which may manifest as subretinal bands and fibrosis, tractional retinal detachment, and permanently impaired vision [111,112].

Miguel et al. reported an incidence of 42% in a 243-patient cohort over 20 years at their center and highlighted that continuous or recurring RD following radiotherapy might indicate ongoing disease activity, which could even serve as a prognostic factor for unsuccessful local control and enucleation [39]. However, a study by Kowal et al. did not find any association between RD and tumor recurrence [113]. Petrovic et al. reported a better prognosis for patients younger than 21 years old compared to adult patients, with the 10-year survival rate being 93% and 65%, respectively, with lower recurrence rates in the juvenile group [79]. Moreover, RD that persisted 6 months after PBT is also identified as a significant risk factor for the development of metastases in the juvenile group [79].

Retinal detachment frequently resolves spontaneously within six months up to a year after radiotherapy; nevertheless, in some instances, it may persist, mainly due to significant inflammation in the treated tumor area and vascular harm caused by radiotherapy [39]. Intraoperative triamcinolone generates regression in 69% of the instances of smaller exudative retinal detachments, although in 12% of such cases, it is accompanied by the side effect of steroid-induced cataract [114]. Although many patients can achieve better visual outcomes with immediate surgical care, non-surgical management is also an option [42]. It has also been shown that exudative retinal detachment was resolved in 73% of patients who had undergone a bevacizumab treatment regimen with a duration of 4 months [43]. Pars plana vitrectomy, sometimes combined with scleral buckling or cataract removal, remains the preferred treatment and can improve visual acuity in most patients [40,41].

#### 3.4.7. Vitreous Hemorrhage

Vitreous hemorrhage is a common finding in patients suffering from uveal melanomas, and the incidence ranges from 4.1% at one year, to 15.1% at five years, and 18.6% at ten years [31,41]. Miguel et al. reported an incidence of 18% in a 243-patient cohort over 20 years at their center [39]. It is usually attributed to the weak adhesion between the retina and sclera caused by the tumor mass due to tumor necrosis or neovascular rupture [31]. It is essential to note that the occurrence of vitreous hemorrhage before melanoma treatment might signal possible tumor invasion through Bruch’s membrane and extensive intraocular tumor dissemination [115]. Radiation therapy debulks the tumor and often lowers the acute risk of vitreous hemorrhage, but it can have an adverse effect on the surrounding retina, causing thinning, as well as ischemia, neovascularization, and fragility on the retinal blood vessels, thereby increasing the risk for late hemorrhage [31,115,116].

The likelihood of developing vitreous hemorrhage after radiotherapy is influenced by various factors, such as pre-existing diabetic retinopathy, shorter tumor-to-optic disc distance, greater initial thickness of the tumor, and rupture of the Bruch’s membrane [61]. Compared to proton-beam therapy, brachytherapy is more frequently associated with vitreous hemorrhage, which can resolve on its own in a few weeks, or it can occasionally recur; therefore, vitrectomy—or in advanced cases, enucleation—may be necessary [1].

Pars plana vitrectomy can treat vitreous hemorrhage directly caused by the tumor without increasing the risk of intraocular, local, orbital, or systemic tumor dissemination [41]. While timely surgical management can lead to improved visual outcomes and facilitate local tumor control or other therapeutic interventions for many patients, observation is also an option [31,41].

#### 3.4.8. Choroidal Complications

Choroidal post-treatment complications are rarely reported, despite the fact that the choroid faces the same vascular changes as the retina, namely blood vessel occlusion, microaneurysms, and choroidal neovascularization. Intravitreal polypoidal choroidal vasculopathy and choroidal folds have also been reported after radiotherapy [29,35].

#### 3.4.9. Optic Neuropathy

Optic neuropathy after radiation is believed to result from demyelination and neuronal degeneration due to glial and endothelial cell damage caused by radiation exposure. This condition frequently leads to irreversible vision loss over time [78]. Plaque size and tumors located subfoveally, juxtapapillary [99,117], and peripapillary [104,118] were identified as factors predictive of optic neuropathy after plaque and proton beam therapy (Figure 2).

The incidence of optic neuropathy differs among different treatment methods for ocular melanoma. When proton beam therapy is used, 68% of patients are affected by optic neuropathy, with a median interval of 17.7 months [105]. This number rises to 89.6% after 60 months [118]. With plaque radiotherapy, 61% of patients experienced optic neuropathy after a median interval of 60 months [99]. Interestingly, LINAC treatment resulted in a much lower percentage of affected patients, with only 14% of them experiencing optic neuropathy after a median interval of 40 months [97]. Likewise, when gamma knife radiosurgery was used, 18.6% of patients were affected by optic neuropathy, with a median interval of 14.9 months [94].

Shields et al. explored treating radiation papillopathy, characterized by a swollen, blood-rich disc surrounded by bleeding, with an injection of triamcinolone acetonide into the eye, observing initial improvements and sustained or better vision in seven patients after 11 months, as clinical symptoms subsided [68]. In the majority of cases, radiation-induced optic neuropathy advances to optic atrophy, resulting in irreversible vision loss.

#### 3.4.10. Tumor-Related Lipid Exudation

Tumor-related lipid exudation (TRLE) is associated with the exudation of lipid and vascular changes in the residual necrotic tumor after irradiation and carries a poor prognosis [61,62,119]. In the literature, TRLE is not always considered as a distinct clinical condition. It is often regarded as a type of radiation-induced retinopathy or mentioned as exudative retinal detachment [61,119]. TRLE is characterized by varying levels of lipid accumulation around the irradiated residual tumor and is often accompanied by varying levels of serous retinal detachment, (Figure 2), [61].

The mean time from radiation to TRLE is 10 months (3–23 months) [62]. Risk factors associated with the development of TRLE are younger age, increased apical height, early occurrence of serous RD after radiation, rupture of the Bruch’s membrane, posterior tumor location, and lack of adjuvant therapy [61,62,119]. It is also associated with a significantly higher incidence of complications after radiotherapy and poor ocular outcome [62,119].

#### 3.4.11. Intraocular Inflammation

Intraocular inflammation is a common occurrence after radiotherapy for uveal melanoma, with around 28% of patients affected up to five years post-treatment [44]. Clinical features usually comprise mild anterior uveitis with cells and flare, increased intraocular pressure, and less commonly, mild anterior vitreous inflammation, possibly due to the release of inflammatory cytokines during tumor necrosis [1,44]. The risk of intraocular inflammation is higher in patients with larger lesions, anterior location of the tumor, or those involving the equator, and patients who receive irradiation over a larger area of the eye [1,44]. To prevent complications, it is important to promptly treat any inflammatory processes that arise following radiotherapy [1,44,120]. Topical steroids and cycloplegics may be used at the onset [44]. Symptomatic treatment is advised in more advanced stages.

#### 3.4.12. Iris Neovascularization—Rubeosis Iridis

Radiotherapy may induce direct and indirect effects on the iris. Some of the direct effects of radiation include atrophy, reduced thickness, and loss of cellularity [51]. Neovascularization is an indirect effect, induced by tumor-related factors or by angiogenic factors produced as a result of inflammation and ischemia in the posterior segment, resulting in rubeosis iridis and neovascular glaucoma [45,51,107]. Careful examination of the iris and anterior chamber angle, prior to dilation, may show early signs of neovascularization. Risk factors linked to rubeosis iridis are increased tumor thickness, anterior tumor location, elevated levels of tumor-related angiogenic factors, increased maximal tumor height, increased internal tumor vascularity, and the disinsertion of a horizontal rectus muscle [4,45].

The incidence of iris neovascularization with or without neovascular glaucoma between 2 to 4 years after iodine-125 brachytherapy has been reported in between 4% and 45% of eyes [121]. Shields et al. [106] have reported that 3% of patients were affected with NVI after plaque radiotherapy and prophylactic intravitreal bevacizumab after a median time of 33 months (4 to 69 months), while Riechardt et al. reported that 20.8% of the patients developed NVI after a mean time of 2 years (range 0.45 to 8.4 years) after PBT [90]. Siedlecki et al. compared the incidence of rubeosis iridis after treatment of uveal melanoma with CyberKnife robotic radiosurgery versus Ruthenium-106 brachytherapy, reporting an incidence of 30.6% versus 5.3%, respectively, at 5 years post-treatment [49].

NVI can be successfully treated with panretinal photocoagulation (PRP) [1,45]. Anti-VEGF therapy has also been used to treat or prevent NVI [46]. Surgical techniques, such as endoresection, have been reported in the literature as an alternative approach [4,46].

#### 3.4.13. Secondary Glaucoma—Neovascular Glaucoma

Secondary glaucoma following radiation treatment can occur through an open or closed angle mechanism [47]. The incidence of secondary glaucoma ranges from 3% to 56%, depending on the type of radiotherapy used [47]. Ruthenium-106 brachytherapy resulted in a lower percentage of affected people in two studies (3–11%) when compared to the results from gamma knife radiosurgery (56%) and CyberKnife radiosurgery (47%) [47,49]. The primary risk factors associated with SG include older age, larger tumor size, anterior location of the tumor, and higher baseline intraocular pressure (IOP) [121]. The first-line therapy for secondary glaucoma is IOP-lowering medical therapy which allows sufficient control, in most cases. Patients unresponsive to conservative therapy may benefit from laser treatment [cyclophotocoagulation (CPC), YAG-iridotomy] [47,48,49] or glaucoma drainage device surgery [50].

Neovascular glaucoma (NVG), a form of secondary glaucoma, manifests as a result of the neovascularization of the iris and the anterior chamber angle [52]. NVG is the major reason for secondary enucleation, and it may occur after all forms of radiotherapy for uveal melanoma [45,47,48,89,99,102,109]. It is believed that the pathogenesis of radiation-induced NVG is based on the release of proangiogenic factors from direct radiation damage to tumor endothelial cells. Additionally, secondary ischemic changes due to injury to the surrounding normal retinal microvasculature may contribute to the development of the neovascularization of the iris [52,122]. It should be noted that the risk for NVG is also influenced by tumor-related factors not related to radiation [47].

Several parameters may contribute to the development or progression of NVG. Tumor thickness [47,59,93,95], higher tumor apical height [109], posterior tumors (located between posterior pole and equator) [47], peripapillary location [47,59,95,123], mushroom configuration [95], volume of posterior segment receiving more that 20Gy [93], Bruch’s membrane rupture [93], higher grade of radiation retinopathy [121], initial retinal detachment [59], and local recurrence [59] are predictive factors reported in the literature.

The reported incidence of NVG varies widely [25,39,48,53,59,95,101,102,103]. Krema et al. reported the incidence of NVG as 8% versus 47% at 50 months post-treatment with I125 brachytherapy versus stereotactic radiotherapy, respectively [124]. Bensoussan et al. studied 492 patients after PBT, reporting an incidence of 27.0% [59]. Kosydar et al. conducted a meta-analysis on the use of Photon-based SRT versus fSRT and reported an overall incidence of 16.8% [125]. It is noteworthy that the time frame also varies greatly, with certain patients experiencing early-onset NVG after just a few months, while others develop late-onset NVG several years after treatment. According to Riechardt et al., the mean time for the diagnosis of NVG after PBT was 2 years [90]. However, the range of time varied widely, from 5 months to 11.6 years [90]. In general, most of the studies report a mean time of NVG peaking between 12–30 months [47,53,90,92,93,95,102,103,104,106,122]. NVG confers a poor prognosis and may lead to enucleation, thus early diagnosis and treatment is of great importance.

Medical therapy to control IOP, anti-VEGF and corticosteroid therapy, panretinal photocoagulation (PRP), glaucoma drainage device surgery, and endoresection have been used in the treatment of NVG (Table 1). Medical therapy cannot control the IOP, in most cases. Intravitreal or intracameral injections of bevacizumab, which are used the lately act by inducing the regression of the neovascularization and decreasing the IOP [4,30,51,52,126]. Panretinal laser photocoagulation remain a widely used therapy for NVG [45,47,52,53]. Other therapies, such as TTT [30,46,54] or endoresection of the residual tumor [30,54,55], have also been used successfully. Efforts have been made for the prevention of NVG using anti-VEGF intravitreal injections (bevacizumab). Shields et al. studied the role of plaque radiotherapy and prophylactic intravitreal VEGF in 1131 patients, with no difference identified between the two groups in the incidence or mean time for developing NVI or NVG [106]. The results of this study remain to be verified or readjusted.

#### 3.4.14. Toxic Tumor Syndrome

Toxic tumor syndrome (TTS) is a severe type of secondary vasculopathy that can occur after radiotherapy for uveal melanoma [47,57]. The term was introduced by Damato et al. to describe the clinical presentation of exudative RD, NVI, and NVG in patients with uveal melanoma who have undergone radiation therapy, (Figure 3), [127]. The pathophysiology of TTS involves the production of proinflammatory cytokines and VEGF by the residual scar after radiation therapy, leading to inflammation and neovascularization in the anterior chamber [1,30,55,57]. Ischemic changes in the retina also contribute to neovascularization [30]. It usually appears between 2 to 5 years after radiation therapy [1,30]. The risk of developing TTS is increased for patients with larger tumor size, retinal and ciliary body invasion, diabetes mellitus, and retinal detachment at the time of diagnosis [57]. If left untreated, TTS has a poor ocular prognosis that often requires enucleation. Both medical and surgical techniques have been used successfully for the treatment and prevention of TTS (Table 1). Endoresection is a promising treatment option that can prevent the development of toxic tumor syndrome by removing the dying tumor, reducing the risk of radiation-induced retinal detachment and neovascular glaucoma [4,51,57,58,59]. Endoresection should be performed within 3 months following radiotherapy for large tumors [57]. Patients who are not eligible for endoresection may benefit from adjuvant surgical methods, such as endodrainage. Although the tumor tissue is not removed during endodrainage, the retina is reattached, which may result in reduced VEGF expression [55,57]. Additionally, other treatments, such as transpupillary thermotherapy, intravitreal injections of anti-VEGF, and intravitreal steroids, can be used to reduce the incidence of TTS [1,30,51,57,58,59,60]. Trans-scleral resection (exoresection) is another option for patients who are unlikely to benefit from other conservative treatments, but it requires a profound hypotensive anesthesia to minimize the risk of intraoperative bleeding [59]. In a study of 12 patients by Konstantinidis et al., symptoms of TTS were resolved after trans-scleral resection [58]. Proper evaluation of timing and indication is crucial for the success of these techniques.

#### 3.4.15. Diplopia and Strabismus

Strabismus and diplopia are the two less-often described side effects after radiotherapy, the incidence of which has been reported between 1.7 and 60% [63,64,65,66,78]. Dawson et al. reported that 16 out of 929 patients (1.7%) who had undergone 125I and 106RU plaque radiotherapy for uveal melanoma developed strabismus or stable diplopia over the following 8 years, while the onset of the latter occurred during the first year in 11 patients [64]. Nagendran et al. evaluated postoperative strabismus in 329 eyes of patients with choroidal melanoma after 106Ru brachytherapy. A total of 41 patients (13.1%) experienced postoperative diplopia, which resolved within 1 month in 18 cases (43.9%), and persistent diplopia developed in only 6 patients (1.9%) [66]. Sener et al. outlined that the incidence of motility disturbance following episcleral plaque brachytherapy with I-125 was 60% (12 out of 20 patients), although diplopia developed in only 2 of them (10%) thereafter [65]. It is important to note that not all patients with strabismus after radiotherapy develop diplopia because of low visual acuity.

The leading causes of strabismus after episcleral plaque brachytherapy include extraocular muscles manipulation, high doses of radiation, and visual impairment. Considerable dissection of the conjunctiva and Tenon’s capsule and mechanical stretching by the plaque leading to relative ischemia of the muscle during the disinsertion period may trigger the development of immediate onset motility disturbances [63,65]. In contrast, a late onset strabismus may arise due to extensive fibrosis and adhesions, radiation scarring, and restriction [64,128]. Most of the cases of strabismus after plaque radiotherapy occur during the first year after the operation. Low visual acuity or decrease more than 6 lines of visual acuity after radiotherapy is a leading cause of sensory strabismus, which can take years after the administration of therapy to develop [63,64,65].

The therapeutic choices for persistent diplopia or strabismus incorporate prisms, botulinum toxin injection, or surgery [63,64,65,66,67]. Along with prism correction, injection of botulinum toxin could be an effective option for the treatment of diplopia in the early postoperative period. Despite the fact that the impact of Botox in restrictive disorders is meager, showing better results in the presence of smaller deviations, it can provide alleviation of diplopia symptoms in patients with good visual acuity for a given period [65]. It is highly advised to wait at least 6 months after the initial surgery prior to performing a strabismus surgery.

#### 3.4.16. Sympathetic Ophthalmia

Sympathetic ophthalmia is an extremely rare, yet potentially vision-threatening, complication of radiation therapy for uveal melanoma that impacts the unaffected eye, and may result in blindness, if suitable therapy is not initiated promptly. It is an autoimmune disorder in which both peripheral blood and vitreous T lymphocytes have been demonstrated to respond to retinal antigen stimulation [69]. In most cases, a penetrating injury or tumor extraocular extension causes disruption of the uveal tract [70]. The incidence after PBT has been reported to be as low as 6.1 in 1000 cases [69]. However, with timely diagnosis and appropriate treatment, visual recovery may be possible. Typical treatment involves a three-day regimen of high-dosage intravenous corticosteroids, followed by oral doses, starting at 1 mg/kg/day, which are gradually reduced, with the potential addition of other immunosuppressive drugs to the treatment plan and the use of topical corticosteroids, in certain instances [69].

#### 3.4.17. Visual Acuity

Vision loss can result from a variety of complications after radiation therapy [100]. Low initial visual acuity, direct macular involvement, and posterior tumor extension are associated with a poor visual outcome, resulting from either immediate or late radiation damage to the macula or optic disc [101,129].

Phacoemulsification for radiation-induced cataracts can be advantageous and safe for patients, particularly in the short-term [107].

Concerning proton beam therapy, about one-third of choroidal melanoma patients with good pretreatment BCVA managed to maintain their visual acuity, while a rapid decline in vision was observed in patients who eventually experienced poor visual outcomes within 6–12 months after treatment [88,130]. Visual loss before therapy is, as expected, associated with a poor visual outcome [129]. Analysis of 5-year data after single-fraction Gamma Knife radiotherapy showed that the majority of patients (84.7%) showed a decline in their vision following treatment, and 13%, 14%, and 36% of eyes exhibited a visual acuity better than 20/40, 20/200, and CF, respectively [131].

Factors like tumor size, tumor proximity to the optic disc or fovea, the amount of radiation absorbed by the macula and optic disc or retina, and the presence of pre-existing retinal detachment are important for visual prognosis [88,101,129,130,131]. The therapy depends on the area of the eye that has received the radiation.

#### 3.4.18. Enucleation Due to Complications

For a long period of time, enucleation was considered the standard treatment for choroidal melanoma. However, after the Collaborative Ocular Melanoma Study demonstrated that radiotherapy was equally effective in extending life; thus, globe-preserving therapies, whenever feasible, are preferred, aiming at a better quality of life [3].

Secondary enucleation may still be necessary in cases of resistance to treatment, local recurrence, or the development of complications such as neovascular glaucoma, persistent exudative retinal detachment, phthisis, functional loss, or ocular inflammation [85,132]. Large tumor size, mainly with a high basal diameter (>18 mm), is the greatest risk factor for enucleation, with the 5- and 10-year eyeball preservation rates being 100% and 96.1% for small tumors and 99.7% and 64.8% for large tumors, respectively. Tumors involving the ciliary body or T4 in TNM are also considered to be high risk [132].

The reported rates of enucleation vary, depending on the type of radiation therapy used, with rates ranging from 3% to 15% for Ru-106 plaque therapy, 1% to 6.8% for I-125 brachytherapy, 4% to 26% for PBT, and 7% to 23% for SRS [91,99,118,133,134,135]. While enucleation may alleviate symptoms and provide topical tumor control, it can adversely affect the patients cosmetically, psychologically, and in terms of quality of life [85].

#### 3.4.19. Recurrences

The incidence of local recurrence following radiation therapy for uveal melanoma varies between 0 and 22% [82,98,117,136]. The two most widely used forms of interventional radiation therapy, such as Iodine-125 and Ruthenium-106 brachytherapy, have a local recurrence rate of 1–10% [101,137,138,139]. Pagliara et al. reported that among 239 patients treated with ruthenium plaque radiation, the estimated local tumor recurrence rates at 12, 24, and 36 months after irradiation were approximately 2.2%, 5.2%, and 8.3%, respectively [81]. The combination of adjuvant transpupillary thermotherapy and ruthenium-106 brachytherapy has been found to potentially decrease the incidence of local recurrence. Proton beams were associated with a local recurrence rate from 3.5% to 15%, while the use of transpupillary thermotherapy presented the widest range of reported local treatment failure rates, ranging from 0% to 55.6% [102,137,138]. Papakostas et al. noted that among 336 patients, local tumor recurrence rates after proton beam radiotherapy were found to be 2.3% at 1 year, 5.0% at 3 years, 7.8% at 5 years, and 12.5% at 10 years [116]. Different types of tumor recurrences may follow, and depending on their growth patterns, they can be classified as marginal, central, diffuse, distant, or extrascleral extensions. Marginal recurrences may be related to an insufficient radiation dose to the tumor border, which can be due to underdosage of the tumor’s edge caused by microscopic disease spread or plaque displacement [137]. Distant recurrences of tumors are uncommon, and they may occur when melanoma cells spread throughout the anterior chamber or when the tumor extends along the ciliary body. Some researchers suggest that distant recurrences may be due to the migration of tumor cells into the exudative retinal detachment [137]. The presence of copy number alterations in chromosomes 3 or 8q in the primary uveal melanoma did not increase the likelihood of local recurrence. However, if a local recurrence did appear, patients with copy number alterations in these chromosomes had a higher risk of disease-specific mortality. Nevertheless, patients with normal copy numbers of chromosomes 3 and 8q had low disease-specific mortality rates, even after experiencing a local recurrence [140]. Identifying and treating tumor progression at an early stage can improve the chances of preserving vision in the affected eye, and may also prevent metastatic spread, in certain cases [138]. However, local tumor recurrence after radiotherapy is linked with higher mortality rates, although it is unclear whether the recurrence is the direct cause of metastatic disease, or simply an indication of more aggressive tumor behavior [60,138].

### 3.5. Quality of Life

Several studies have evaluated the impact of radiation therapy on the quality of life (QoL) in patients with uveal melanoma. The diagnosis of ocular melanoma compromises QOL, which is additionally impaired by subsequent treatment. Regular assessments of the quality of life can help identify at-risk patients, provide psychosocial treatment, and improve patient satisfaction [141]. The European Organization for Research and Treatment of Cancer (EORTC) created a 30-item QOL survey, the QLQ-OPT30, to assess patients diagnosed with uveal melanoma. This survey is often used together with QLQ-C30, a generic health-related QOL questionnaire that is widely used for various types of cancer [142,143]. The type of treatment or the location of ocular melanoma do not seem to have an impact on the health-related quality of life. Moreover, the selection of treatment for ocular melanoma does not appear to significantly affect the quality of life in the long run, as there were no significant differences in the quality of life of patients who underwent different methods of radiotherapy or enucleation [142,144,145,146,147]. Hope-Stone et al. also showed that patients experienced a QoL similar to that of the general population 6 months after treatment [148].

Various factors, mainly low final visual acuity, extraocular extension of the tumor, and high IOP, have been associated with a decrease in QOL [149]. Shortly after radiotherapy, patients are often more anxious about the possibility of local tumor recurrence and experience increased discomfort due to diplopia and headaches [144,150,151]. Additionally, patients who undergo enucleation and those who receive conservative therapy are equally likely to report concerns regarding local tumor recurrence [144]. Suchocka-Capuano et al. noted that more than half of 69 patients had a moderate rate of anxiety before starting treatment, which significantly decreased a month later [146]. Women and younger patients are more vulnerable to anxiety, although there is no significant gender-based difference regarding depression [144,148]. Furthermore, anxiety levels tend to diminish during the first year after treatment, particularly in younger patients, while remaining stable in older patients [143,148,151]. Patients with monosomy 3 appeared to be more depressed than others at every time point [148]. Lower QoL scores were observed in patients who developed ocular symptoms after receiving radiation therapy for uveal melanoma, emphasizing the importance of managing such symptoms, including pain or redness, to achieve better QoL outcomes [144,152].

## 4. Conclusions

In conclusion, radiation therapy is a valuable treatment option for uveal melanoma, but it can result in various vision-threatening ocular complications. The key to managing these complications lies in early detection, the establishment of a treatment plan, and expert medical care. The development of preventative measures and the advancement of therapeutic options have improved patient outcomes. Globe-preserving therapies, such as proton beam radiotherapy, plaque brachytherapy, and stereotactic radiotherapy, have been successfully in recent last decades. By understanding the pathogenesis, risk factors, and management of complications associated with radiation therapy, patients and physicians can work together to achieve the best possible outcome while preserving ocular function and quality of life.

## Figures and Tables

**Table 1:** Therapeutic approaches to the most common complications following radiotherapy for uveal melanoma.

Complication	Available Therapeutic Approaches
Ocular Surface and Ocular adnexa complications	Artificial tears, topical corticosteroids, and antibiotic treatment [20]
	Reconstructive surgery, tarsorrhaphy, or gold weight implantation [20]
	Contact lenses, autologous serum eye drops, conjunctival flaps, amniotic membrane transplantation, cyanoacrylate gluing [20,21]
	Lamellar or penetrating keratoplasty [20,21]
Scleral Necrosis	Observation Artificial lubrication, gels or ointments, like prednisolone acetate 1% [22,23,24,25,26,27,28]
	Surgical techniques [22,23,24,25,26,27,28] Tissue glue Amniotic membrane transplantation Conjunctival graft/flap Scleral patch graft Vital Tenon’s fascia transposition Dermal patch graft Hyperbaric oxygen therapy Enucleation
Scleritis	Systematic [28,29] Corticosteroids NSAIDs
	Topical [28,29] Cycloplegics Beta-blockers Corticosteroids NSAIDs
Radiation Induced Cataract	Phacoemulsification [30,31,32]
Radiation Maculopathy	Intravitreal anti-VEGF therapy (bevacizumab, ranibizumab, and aflibercept) [33,34,35,36,37]
	Intravitreal corticosteroids (triamcinolone acetonide and dexamethasone implant) [33]
	Sub-tenon triamcinolone acetonide
	Intravitreal fluocinolone acetonide implant [38]
Retinal Detachment (RD)	Observation [39]
	Pars plana vitrectomy [40,41]
	Scleral buckles [40,41]
	Intravitreal triamcinolone, bevacizumab [42,43]
Vitreous Hemorrhage (VH)	Observation [31,41]
	Pars plana vitrectomy (in case of recurrence or complications) [31,41]
Ocular Inflammation	Topical corticosteroids and cycloplegics [1,44,45]
Iris Neovascularization (NVI)	Panretinal photocoagulation (PRP) [1,45]
	Anti-VEGF therapy [46]
	Surgical techniques—endoresection [4,46]
Secondary Glaucoma (SG)	IOP-lowering medical therapy
	Laser treatment [47,48,49] Cyclophotocoagulation (CPC) YAG-iridotomy
	Glaucoma drainage devices [50]
Neovascular Glaucoma (NVG)	IOP-lowering medical therapy and glaucoma drainage devices
	Intravitreal or intracameral bevacizumab [4,30,51,52]
	Panretinal photocoagulation (PRP) [45,47,52,53]
	Transpupillary thermotherapy (TTT) [30,46,54]
	Surgical techniques [30,54,55,56] Endoresection Endodrainage
	Enucleation
Toxic Tumor Syndrome (TTS)	Endoresection [4,51,55,57,58,59]
	Intravitreal anti-VEGF and steroids [1,30,51,59,60]
	Endodrainage [55,57]
	Transpupillary Thermotherapy (TTT) [1,30,51,59,60]
	Exoresection [59]
Tumor-Related Lipid Exudation (TRLE)	Transpupillary thermotherapy (TTT) [61]
	Anti-VEGF therapy [62]
	Local resection [62]
Diplopia and Strabismus	Prisms Botulinum toxin injection Strabismus surgery [63,64,65,66,67]
Optic Neuropathy	Intravitreal injection of triamcinolone acetonide (4 mg/0.1 mL) [68]
Sympathetic Ophthalmia	Intravenous corticosteroids, followed by oral administration, starting at 1 mg/kg/day, with a gradual tapering [69]
Visual Acuity	Treatment depends on the part of the eye that has received the radiation

**Table 2:** Current clinical trials for the prevention or treatment of ocular complications after radiotherapy.

Trial	Identifier
Dexamethasone Implant for Retinal Detachment in Uveal Melanoma	NCT04082962
Influence of Oral Treatment with Citicoline for the Prevention of Radiation Optic Neuropathy in Patients Treated for Uveal Melanomas With Proton Beam Therapy	NCT01338389
Endoresection of the Tumor Scar or Transpupillary Thermotherapy for the Treatment of Large Uveal Melanomas (Endoresection-Laser)	NCT02874040
Prevention of Neovascular Glaucoma by Intravitreal Injections of Anti-VEGF in Patients Treated with Proton Therapy for a Large Choroidal Melanoma (PROTECT)	NCT03172299
Safety and Efficacy of Silicone Oil Tamponade for Surgical Attenuation of Radiation Damage in Choroidal Melanoma	NCT01460810
